# Age-related changes in Folliculogenesis and potential modifiers to improve fertility outcomes - A narrative review

**DOI:** 10.1186/s12958-022-01033-x

**Published:** 2022-11-17

**Authors:** Ecem Esencan, Gabriela Beroukhim, David B. Seifer

**Affiliations:** Yale School of Medicine, Department of Obstetrics, Gynecology, and Reproductive Sciences, New Haven, CT USA

**Keywords:** Ovarian aging, Folliculogenesis, Antimullerian hormone, Vascular endothelial growth factor, Neurotropins, Insulin-like growth factor, Vitamin D, Coenzyme Q, Dehydroepiandrosterone

## Abstract

Reproductive aging is characterized by a decline in oocyte quantity and quality, which is directly associated with a decline in reproductive potential, as well as poorer reproductive success and obstetrical outcomes. As women delay childbearing, understanding the mechanisms of ovarian aging and follicular depletion have become increasingly more relevant. Age-related meiotic errors in oocytes are well established. In addition, it is also important to understand how intraovarian regulators change with aging and how certain treatments can mitigate the impact of aging. Individual studies have demonstrated that reproductive pathways involving antimullerian hormone (AMH), vascular endothelial growth factor (VEGF), neurotropins, insulin-like growth factor 1 (IGF1), and mitochondrial function are pivotal for healthy oocyte and cumulus cell development and are altered with increasing age. We provide a comprehensive review of these individual studies and explain how these factors change in oocytes, cumulus cells, and follicular fluid. We also summarize how modifiers of folliculogenesis, such as vitamin D, coenzyme Q, and dehydroepiandrosterone (DHEA) may be used to potentially overcome age-related changes and enhance fertility outcomes of aged follicles, as evidenced by human and rodent studies.

## Introduction

Female fertility rates increase after puberty and peak in the early 20s [1]. This is followed by a decline in fertility thereafter, which is salient after age of 35 years [1]. The process of reproductive aging is characterized by a quantitative and qualitative deterioration in ovarian reserve, associated with increases in aneuploidy and higher rates of miscarriage [2, 3]. Therefore, biological age is a critical factor in reproductive success.

Increased participation of women in education and the work force has been paralleled by a significant delay in childbearing [2]. Consistent with later attempts at pregnancy, female fertility rates have significantly declined, and prevalence of diminished ovarian reserve (DOR) and use of donor eggs among the population undergoing assisted reproductive technologies (ART) have increased [2]. Ovarian aging is accompanied by a quantitative deterioration and qualitative decline in ovarian reserve with an increase in oocyte aneuploidy, reduced embryo quality and increased miscarriage rates [2, 3]. In addition, among those with DOR, the granulosa cell (GC) layer of the growing follicles secrete less estradiol and anti-mullerian hormone (AMH), they require higher doses of gonadotropins, leading to a poorer response to ovarian stimulation thus necessitating use of donor eggs among the population [4]. Given these demographic and socioeconomic changes and their impact on fecundity and response rate to ART, there is increasing interest in better understanding the biological mechanisms of ovarian aging and methods to potentially overcome or delay aging of the finite pool of follicles that women are born with.

For this review, we searched various combinations of: follicle, oocyte, follicular growth, cumulus cells (CC), follicular fluid (FF), aging, fertility in PUBMED through June 2022. We included relevant full text, English language articles in this manuscript. We aim to briefly introduce folliculogenesis and to explore the mechanisms related to ovarian aging. We then discuss the role of specific small molecules in ovarian aging and how such molecules can be used to detect DOR and/or to increase fertility outcomes of aging follicles. We aim to provide a comprehensive review of changes in folliculogenesis with aging, as well as the various molecules and pathways involved in regulation of aging, including AMH, vascular endothelial growth factor (VEGF), neurotropins, insulin-like growth factor 1 (IGF1), and mitochondrial function/dysfunction. We also review how the usage of vitamin D, coenzyme Q, and dehydroepiandrosterone (DHEA) may reduce the effect of aging in oocytes and lead to enhanced fertility outcomes.

## Background

**Fig. 1:**
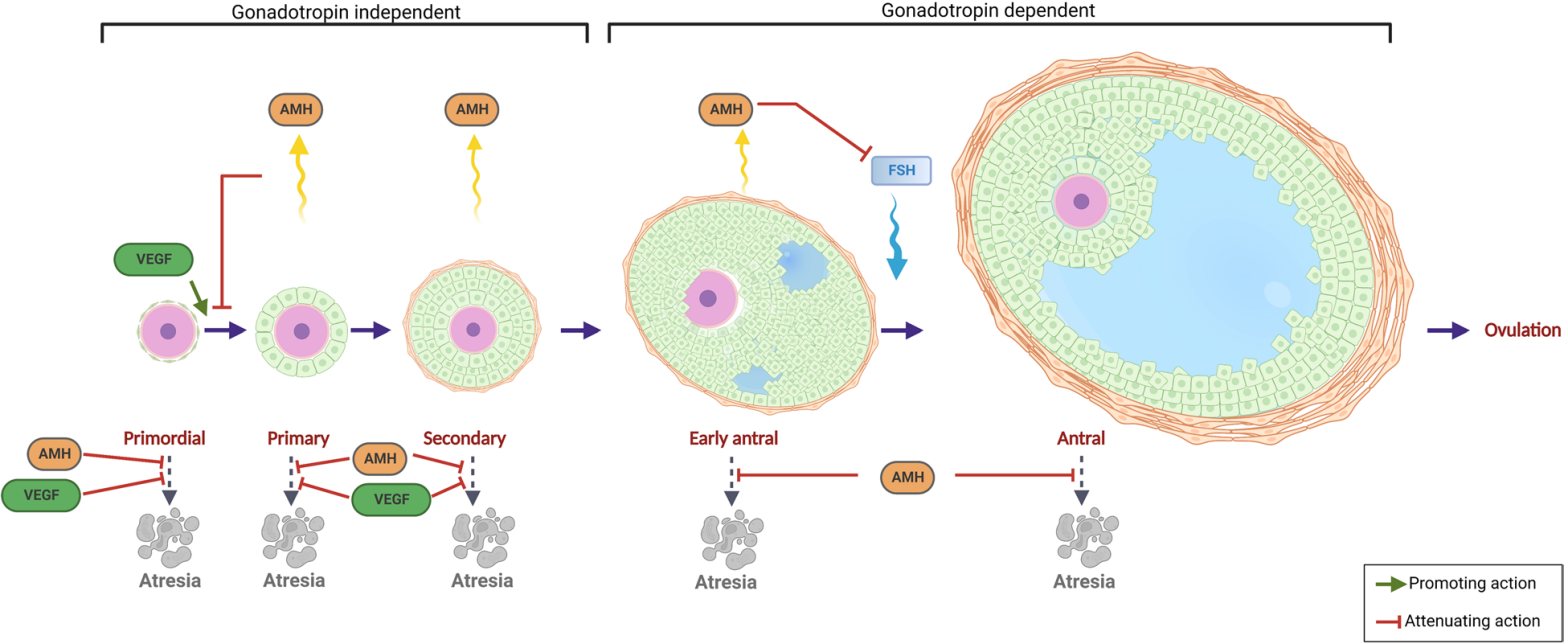
Folliculogenesis: Primordial follicles are surrounded with a single layer of squamous granulosa cells. With primordial activation, primordial follicles are recruited into folliculogenesis, initially becoming primary follicles with a single layer of cuboidal granulosa cells. With replication, granulosa cells form layers surrounding the oocyte, thus forming the secondary follicle. The early/small antral follicle is comprised of multiple layers of granulosa cells, which are differentiated into cumulus cells that immediately surround the oocyte as well as small pockets of antrum. The antral follicle is characterized by a large antrum containing follicular fluid, and is ready to be ovulated out of the ovarian cortex. Follicles can undergo atresia at any stage of folliculogenesis. AMH is secreted by preantral follicles and control primordial follicle recruitment as well as follicular atresia. In comparison, VEGF stimulates recruitment of primordial follicles to become primary

Women are born with a finite pool of primordial follicles and this number declines from approximately 2 million to 400,000 by the time women reach menarche [5]. During female reproductive years, a cohort of primordial follicles is recruited monthly in order to grow a single dominant follicle and ovulate its oocyte competent for fertilization and embryo formation. In primordial follicles, the dormant oocyte is arrested at prophase I and is surrounded by a single layer of flattened GCs [6]. Primordial follicle recruitment leads to activation and maturation of the oocyte, as well as proliferation and differentiation of surrounding GCs, leading to the formation of primary follicles, which are characterized by a single layer of cuboidal GCs surrounding the oocyte. GC layers continue to proliferate in secondary follicles. They differentiate into CCs, which are cells contiguous with the maturing oocyte [7, 8] and are also important in recruitment of androgen producing theca cells (later segregated into theca interna and externa layers) from progenitor pool at ovarian mesenchyme and mesonephros [9]. Until the formation of the preantral follicle, folliculogenesis is gonadotropin independent and is a paracrine signaling unit comprised of multiple locally active growth factors and small molecules secreted by GCs of the early follicle [10] (Fig. 1).

With growth and differentiation of GCs, maturing follicles eventually express gonadotropin receptors and acquire responsiveness to gonadotropins, first to follicle-stimulating hormone (FSH) then to luteinizing hormone (LH). In this gonadotropin dependent phase of folliculogenesis, follicles become hormone secreting units. FSH enables GC growth, promotes estradiol production, and enables dormant follicle selection [11], while LH is responsible for androgen secretion from cholesterol, completion of meiosis I in the oocyte, germinal vesicle breakdown [12–14] and subsequent progression to metaphase II [15]. Ovulation marks the end of folliculogenesis with production of a mature oocyte capable of fertilization [16–18].

Autocrine and paracrine intraovarian factors are influential in both gonadotropin-independent and -dependent phases of folliculogenesis [19]. Within each follicle crosstalk between CCs and the oocyte occurs through gap junctions, formed with oocyte-provided connexin 37 and CC-expressed connexin 43 [20]. This process enables nutrient exchange between two compartments of the cumulus-oophorus complex [20] and is crucial for healthy follicle formation [21–23]. Quality of oocytes are therefore paralleled by proper CC functioning [24]. Through these junctions, oocyte receives pyruvate, amino acids, and nucleotides as well as provides CCs with growth factors, such as bone morphogenetic protein 15 (BMP15) and growth differentiating factor 9 (GDF9), which are obligatory for proper folliculogenesis [25–29]. GDF9 is responsible for GC proliferation and differentiation to CCs [30]; its levels continuously rise during folliculogenesis until ovulation [27] and absence of GDF9 leads to arrest at the primary follicular stage with subsequent follicular atresia and infertile mice [25]. Knock out of *Bmp15* also disrupts folliculogenesis leading to subfertility in mice [28] and infertility in sheep [29].

Throughout the highly regulated and well-orchestrated folliculogenesis process, multiple follicles are arrested at different stages and go through irreversible atretic degeneration with increasing pace after the mid-30s [5, 31]. With aging, in addition to quantitative decline, follicles go through progressive deterioration in their function and in their capacity to form an oocyte capable of fertilization.

Aging related disruption in folliculogenesis is attributed to changes that most likely occur simultaneously and include bioenergetics dysfunction leading to increased oxidative stress and shortened telomere length in aged oocytes obtained from antral follicles, and decreased DNA repair capacity with increased mutations through loss of chromosome cohesion and spindle aberration leading to meiotic errors during folliculogenesis [32, 33].

Such age-related disruptions are not limited to the oocyte but also affect the GCs and CCs [34, 35]. In addition to the number of follicles, the amount of GCs surrounding the oocyte also diminish with age due to increased GC apoptosis [36], which is accompanied by altered production of locally active growth factors [37], attenuated proliferation [38] and disrupted steroidogenesis [39].

## Role of intraovarian paracrine and endocrine molecules in folliculogenesis and the aging follicle

### AMH is a critical intra-ovarian regulator of the gonadotropin independent phase of folliculogenesis and an indirect marker of the primordial follicular pool which decreases with age

AMH also known as Mullerian inhibiting substance, is a member of the TGFβ superfamily of peptides that acts through its specific receptor, AMHR2. It was first described for its role in fetal sexual differentiation in 1950s by Professor Alfred Jost. In the male fetus, around 8 weeks of gestation AMH is secreted by Sertoli cells of testis and binds to AMHR2 located at the surface of Mullerian duct leading to its regression by apoptosis [40, 41]. A mutation of the AMH gene located on chromosome 19q13 or a mutation of the AMHR2 gene located on chromosome 12q3 leads to Persistent Mullerian Duct Syndrome, an autosomal recessive disorder characterized by the presence of a uterus and fallopian tubes in an otherwise male appearing individual [42, 43]. In female fetus, AMH expression in the ovary starts at approximately 36 weeks of gestation, peaks in the mid-20s, and continues to decrease until menopause [44–46]. In the absence of AMH in early female fetal development, the Mullerian duct persists and form the fallopian tubes, uterus, cervix, and upper one-third of vagina. In the ovary, *Amh* and its receptor *Amhr2* are mainly expressed in GCs of maturing follicles with altering expression levels in different stages [47]. *AMH* expression starts in GCs of primary follicles, gradually increasing and reaching the highest level in small antral follicles less than 6 mm in size [44, 48]. In antral follicles, the majority of AMH is secreted by CCs [47]. In atretic follicles and the corpus luteum (CL), AMH levels are undetectable [47]. *AMHR2* expression mirrors the *AMH* expression pattern and is also secreted in smaller amounts by theca cells of preantral and antral follicles [47].

In rodents, AMH has been shown to act as a gatekeeper of finite primordial follicular reserve by inhibiting the initial gonadotropin independent recruitment of the follicles and sustaining their dormant state [45, 49] (Fig. 1). *Amh* knockout mice models demonstrated increased number of maturing follicles and smaller pools of primordial follicles [50, 51]. Human studies on primordial reserve using ovarian cortical biopsies are still controversial regarding AMH’s role in inhibiting primordial follicle assembly [47]. Conversely, overexpression of AMH during fetal gonadal development negatively impacts normal ovarian and follicular pool development [52]. AMH is also crucial in cyclic antral follicle selection. During gonadotropin-independent and in early gonadotropin–dependent phases of follicular development, AMH has an inhibitory role on the growing follicle [53]. For example, *Amh* knockout mice models have been shown to recruit significantly more growing follicles with ovarian stimulation compared to the control group [54]. This is suggested to be achieved through halting sensitivity to FSH and disrupting steroidogenesis through AMH’s down-regulatory effect on the expression of aromatase (*Cyp19a1*) and the LH receptor (*Lhcgr*) as demonstrated in rodent and porcine follicles [47] (Fig. 1). This is further supported by Hayes et al. who established that in vivo AMH treatment of mouse, decreases *Cyp19a1*, *Lhcgr* and steroidogenic acute regulatory protein (*Star)* expression [55]. Decreased estrogen production through inhibited aromatase, contributes to AMH’s inhibitory effect on follicular recruitment [49].

The regulation of AMH and expression of its receptors are complex and not yet fully understood. Among the gonadotropins, low concentrations of FSH are shown to promote *Amh* expression in vitro mouse and human studies [56, 57]. Conversely, estrogen inhibits both *Amh* and *Amhr2* expression in in vitro and in vivo studies [58]. In in vitro studies, addition of steroids, specifically estrogen, to FSH containing culture medium halts FSH’s positive effect on AMH expression [59, 60]. This phenomenon is supported by in vivo studies of ovarian stimulation with FSH which upregulates estrogen production which in return inhibits *Amh* and *Amhr2* expression [61, 62]. Similar to estrogen, LH also has an inhibitory role on *Amhr2* expression [58, 63]. The effect of androgens on AMH and AMHR2 expression has yet to be clearly defined.

In addition to systemic gonadotropins, an expanding list of intraovarian factors including BMPs also regulate early follicle growth as well as *Amh* expression. Most in vivo and *vitro* studies show enhancing effect of most BMPs on *Amh* and *Amhr2* expression. Among many intraovarian factors, BMP15 is the most well studied. BMP15 alone increases both *Amh* and *Amhr2* expressions [64, 65]. GDF9 alone hasn’t been shown to repress or stimulate AMH expression, however in the presence of BMP15, it augments BMP15’s stimulatory effect on AMH expression [64, 66–68]. Recombinant AMH also increases expression of GDF9 and BMP15 promoting better oocyte quality [69]. Others such as BMP2, BMP4 and BMP6 have also been shown to upregulate *Amh* expression in in vitro studies [65, 70, 71].

The role of environmental endocrine disruptors and metabolic factors on *Amh* expression and folliculogenesis remain under investigation. However, initial studies support that insulin can upregulate *Amh* expression in a dose dependent manner [72]. Other growth factors such as, VEGF and tumor necrosis factor alpha (TNFa) inhibit *Amh* mRNA levels [73]. AMHR2 levels have also been studied and demonstrate that leptin represses its expression [74], whereas IGF1 and VEGF upregulate its expression [73, 75]. Environmental factors such as abnormal vitamin D levels, oral contraceptive pills, and smoking have also been shown to alter AMH expression levels [49].

**Fig. 2:**
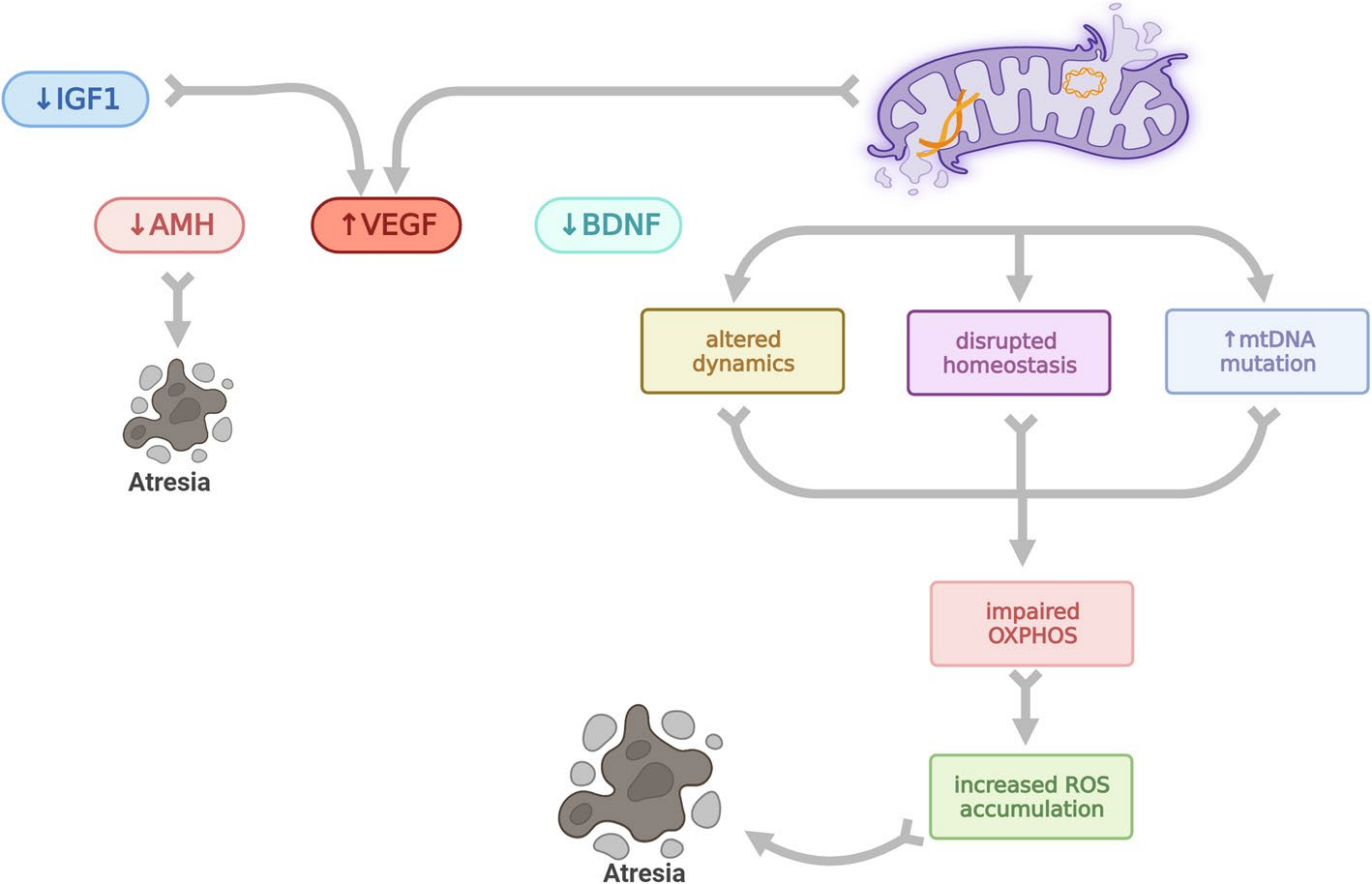
Age related changes of intraovarian small molecules and mitochondrial function within the follicle: Aging is related to decreased IGF1, decreased AMH, decreased BDNF, increased VEGF levels as well as mitochondrial dysfunction. Altered mitochondrial dynamics, disrupted mitochondrial homeostasis and increased mtDNA mutations are age related changes that occur within mitochondria and lead to impaired oxygenation-phosphorylation. Impaired oxygenation-phosphorylation leads to increased ROS accumulation which leads to follicular atresia

AMH is postulated to be anti-apoptotic on GCs with a protective role against follicular atresia [55] (Fig. 1). Although a casual pathway was not determined, in natural IVF cycles, AMH level was noted to be elevated while atretic antral follicle number was reduced [76] The rate of decrease in AMH over time is mirrored by an age-related rate of atresia (Fig. 2). AMH null mice exhibit increased number of atretic follicles in their ovaries [77] and *Amh* knockdown in macaque ovaries results in impaired secondary follicle survival [78]. Consistently, treatment with AMH reduces atretic follicle number in prepubertal [79] and adult mice [55] as well as cryopreserved mouse ovarian tissue [80] with higher primordial and primary follicular count. AMH, also a marker of ovarian reserve that was initially described by Seifer et al. in 2002, [81] is used clinically worldwide as a noninvasive, reproducible, and rapidly interpretable method associated with a high positive predictive value and low negative predictive value for number of retrieved oocytes. Due to its role in regulating the rate of follicle recruitment [82, 83] and low intracyclic variability with circulating gonadotropin levels [84], AMH is often utilized for fertility preservation counseling and individualizing ART treatment. Serum AMH levels are also used for prediction of menopause onset and for minimizing the risk of potentially life-threatening ovarian hyperstimulation syndrome in women undergoing ovulation induction [48]. In addition, in follicular fluid AMH levels can be used to determine GC apoptosis rate in ART cycles [76].

### VEGF is crucial in gonadotropin-independent folliculogenesis through promotion of angiogenesis and is increased in aging follicles

VEGF is a potent angiogenic factor expressed in a variety of tissues, and plays a role in the complex autocrine, paracrine and endocrine regulation of the gonadotropin-independent phase of folliculogenesis [85]. VEGF, especially VEGFa, and its receptors, VEGFR1 and VEGFR2 are regulatory factors in mammalian ovarian folliculogenesis [86–89], and their expression levels increase as follicles mature [90, 91]. In the ovarian cortex, small preantral follicles do not possess their own blood supply, but rather receive their nutrition and oxygenation through passive diffusion from stromal tissue. During follicular maturation, starting with secondary follicles, outer stromal cells surrounding the oocyte differentiate into theca cells [92]. The inner theca layer, which is separated from the granulosa cell layer via a basement membrane, form a capillary network for the follicle that is essential for its growth [93, 94]. Injection of VEGF directly into the ovarian blood supply can positively affect angiogenesis, increase the number of primary and secondary follicles and reduce follicular atresia [87, 95] (Fig. 1). In cows, VEGF administration increases the number of secondary follicles [89]; and in primates, VEGF inhibitor administration decreases endothelial cells in secondary follicles and inhibits antral follicle formation [96]. VEGF has been shown to promote primordial pool activation in in vitro studies [97]. VEGF expression in the follicle progressively enhances maturational development from primary to preovulatory stages and is positively correlated to the diameter of the follicle [85]. In rodents, *VegfR* mRNA has been identified specifically in theca cells of secondary follicles [89]; and in humans, *VEGFa* and *VEGFR* gene expression and protein have been detected in theca cells, granulosa cells and in oocytes [98]. Thecal angiogenesis and increased vascular permeability through VEGF are thought to be crucial for follicular growth and for antrum formation by providing nutrients and oxygenation to the follicle as well as securing adequate supply of gonadotropins, steroid precursors and other folliculogenesis regulators to the developing follicle [94]. This phenomenon has been postulated to be decisive for follicular dominance with the dominant follicle containing more abundant vasculature in its theca layer compared to others in the same cohort [99].

A robust capillary network is also essential for formation of a functional CL. The mid-cycle LH surge leads to GC *Vegf* expression [100], proteolytic activity, and degradation of the basement membrane leading to VEGFa release into the GC layer, and new capillary formation in the developing CL [101]. Administration of a GnRH antagonist leads to decreased *Vegf* expression in CL of primate ovaries by inhibiting the LH surge [102].

Gonadotropins and other angiogenic factors secreted by GCs can enhance VEGF expression within the follicle [103, 104]. In addition, some intraovarian regulators such as theca derived BMP7 have been demonstrated to induce *VEGFa* mRNA and protein expression and increase endothelial cell sensitivity to VEGF in human GCs and enhance follicular angiogenesis [105]. Cytokines such as interleukin 6 (IL6), IGF1 and fibroblast growth factor 2 (FGF2) have been identified with their pro-angiogenic role in ovary, inducing *VEGF* expression [106–108].

In the setting of inadequate theca vasculature formation, hypoxia may lead to altered oocyte metabolism and decrease in intracellular pH in the oocyte which might both deteriorate spindle formation and organization leading to chromosomal abnormalities as well as enhanced follicular atresia [109]. For example, severe reduction in dissolved oxygen in FF among patient aged 25–37 years old, is associated with an increased number of chromosomal abnormalities on meiotic spindles in metaphase II [110]. With aging, VEGF levels in FF increase among both natural and in vitro fertilization (IVF) cycles [37, 111–115] (Fig. 2). This age-related increase in VEGF levels may be secondary to selective elevation of gonadotropins in older reproductive age women as well as a compensatory response to follicular hypoxia and decreased energy synthesis in the setting of disrupted mitochondrial function [10].

### Mitochondrial function and dynamics are regulators for folliculogenesis and are compromised with aging

Within the follicle, glucose is the key energy source [116] and is mainly metabolized by the supporting CCs [117] which are in direct communication with the oocyte through protrusions through the zona pellucida [118]. Through this intercellular junction, oocytes receive pyruvate, an end metabolite of glycolysis, amino acids, nucleotides, glutathione and local growth factors [119] and provide CCs with oocyte derived regulators of CC gene expression [120]. Normal functioning mitochondria are essential in the cytoplasm of oocytes, not only for their role in utilization of pyruvate and adenosine triphosphate (ATP) production through oxidative phosphorylation (OXPHOS) [121], but also for apoptosis [122], amino acid metabolism, phospholipid synthesis and calcium signaling [123, 124].

Mitochondria are crucial in all stages of folliculogenesis and follicular aging. Mitochondrial number, function, and dynamics have been shown to be altered with advancing age in all compartments comprising the follicle [34, 125]. Aging has been associated with disruption in various mitochondrial functions including amount of mitochondrial DNA (mtDNA) mutations, impaired OXPHOS, accumulation of reactive oxygen species (ROS), decreased mitochondrial dynamics, as well as disruption of pathways involved in mitochondrial homeostasis [126–130]. Optimal mitochondrial function is important for normal chromosome distribution. Thus, mitochondrial dysfunction also contributes to chromosomal abnormalities and failure to extrude the first polar body in the oocyte through meiotic division errors, which occur at higher incidence in women with advanced age [131]. Moreover, dysfunctional mitochondria may lead to decreased ATP production and increased oxidative stress within the oocyte due to its inability to balance ROS production. Babayev et al. demonstrated that older mice have smaller mitochondria in oocytes obtained from primary follicles, increasing ROS with induced cellular stress in their mature oocytes [132]. Not only in oocytes but in CC obtained from poor ovarian response patients and women over 38 years old, follicular mitochondrial function has been shown to be severely impaired [133–135]. The impaired mitochondrial function in oocyte and age related ROS accumulation are directly related to decreased oocyte quality [136] with arrested maturation, halted fertilization, compromised embryo development and increased embryo aneuploidy [131] (Fig. 2).

Mitochondria are dynamic organelles that adjust to cellular needs through fission and fusion [137]. *Drp1* gene has been noted to regulate fission enabling one mitochondrion to divide into two, and it activates mitophagy (mitochondrial autophagy) mainly through the PINK1/Parkin pathway for removal of abnormal mitochondrial content. In return, fusion enables diluting damaged mitochondrial content and improves the mitochondrial pool quality. *Opa1*, *Mfn1* and *Mfn2* are important regulators of mitochondrial fusion [1]. While *Opa1* controls inner mitochondrial membrane fusion, *Mfn1* and *2* are responsible for outer membrane fusion [138]. Within the oocyte, optimal mitochondrial dynamics are altered with aging as evidenced by several mice studies [132, 139]. Global knockout of *Opa1*, *Mfn1*, *Mfn2* or *Drp1* in mice have been shown to be lethal in embryogenesis [140–142]. Oocyte specific knock out of Drp1 in mice demonstrated that fission of mitochondria is important for follicular maturation and ovulation [143]. Zhang et al. demonstrated that lack of MFN1 specifically at mice oocytes leads to infertility through accelerated follicular pool depletion secondary to defective follicular maturation with arrest at secondary stage as well as impaired oocyte maturation and increased apoptosis [144]. Lack of another fusion protein, MFN2 in oocytes also lead to abnormal follicle development and impaired oocyte maturation through shortened telomere length leading to subfertility and DOR in female mice, mimicking aging related changes [145].

Mitochondria are known to carry their own double stranded mtDNA; its content increases during oogenesis though is constant during oocyte maturation, fertilization, and blastocyst formation [146–148]. With increasing age, the body’s antioxidant capacity decreases and dormant follicles are exposed to accumulated ROS for prolonged periods of time leading to potential mutations in mtDNA and deficits in adequate functioning [149]. Given the lack of histones and an effective DNA repair system and its proximity to ROS produced within mitochondria, mtDNA is postulated to be more susceptible to oxidative damage than nuclear DNA [150]. It has been shown that mtDNA deletions, which cause deficits in proper OXPHOS may be responsible for increased GC apoptosis within aged follicles [151, 152]. Specifically, mt4944, the most commonly studied mtDNA mutation, is noted to accumulate in aging ovarian tissue [153] and within both granulosa cells [135] and the oocyte [154]. However, it is interesting that the studies assessing abnormal mtDNA number, whether high or low, and its impact on oocyte quality, fertilization, and implantation potential have been conflicting [155–161]. In one study, Fragouli et al. demonstrated that increased mtDNA is detected in embryos obtained from advanced maternal age patients, euploid embryos that failed to implant, and aneuploidy embryos regardless of maternal age [162]. Whereas, Diez-Juan et al. did not observe any difference in mtDNA content within embryos obtained from old and young females [163]. In two other studies conducted by Victor et al. and Klimczak et al., mtDNA content was not significantly altered with age, chromosomal aneuploidy, or implantation potential [158, 161].

In addition, mitochondria have an unfolded protein response (mtUPR) system, which enables a response to cellular stress and enhances mitochondrial hemostasis with maintaining the balance of a healthy mitochondrial pool. It identifies and eliminates misfolded proteins and promotes appropriate protein folding within the mitochondria [164]. mtUPR also promotes coenzymeQ10 (CoQ10) synthesis, glycolysis, and mitochondrial dynamics [1]. In an aging mouse model, expression of *Hspd1*, a mtUPR gene was downregulated in oocytes obtained from old mice with prior proven fertility [132]. Disruption of this mtUPR system leads to mitochondrial malfunction. Global deletion of *Clpp*, a mitochondrial protease involved in mtUPR system, leads to infertility in female mice with accelerated ovarian reserve depletion consistent with the DOR phenotype (Fig. 2). These mice demonstrate a decreased number of follicular responses to gonadotropin stimulation and fail to make blastocysts. This is believed to be secondary to impaired mitochondrial functioning, disrupted OXPHOS, and ROS accumulation within the oocyte [165]. mtDNA content on oocytes obtained from CLPP deficient mice also noted to be higher signaling mitochondrial distress [165]. They also noted a higher mtDNA content in oocytes. In another study with targeted *Clpp* deletion solely in cumulus cells, expression of mitochondrial dynamics and cellular metabolism genes were altered and apoptosis in cumulus cells was increased with a decreased number of cumulus cells within the follicle [166].

### IGF1 is an important paracrine modulator of gonadotropin-dependent folliculogenesis and its levels in FF decrease with age as well as DOR

The IGF system consists of IGF1, IGF2, their specific receptors (IGF1-R and IGF2-R), and IGF binding proteins (IGFBPs), which are inhibitors of IGF expression. IGF1 and IGF2 are growth factors that are mainly secreted by hepatic cells through growth hormone (GH) influence [167]. They are potent mitogens, promoting DNA synthesis, involved in cell proliferation and differentiation [168, 169] and are known to suppress apoptosis [170]. All members of IGF system have also been identified in the mammalian ovary [171–173]. They are primarily synthesized in GCs of preovulatory follicles [174, 175] and are important intraovarian modulators of folliculogenesis [176]. In an autocrine manner, IGF1 and IGF2 enhance GC proliferation and differentiation [177, 178], promote steroidogenesis by acting synergistically with gonadotropins [179–181], and prevent follicular atresia, enhancing survival of small growing follicles [172, 182]. In in vitro grown rat GCs, supplementation of IGF1 increases aromatase activity, amplifies FSH induced estrogen production [174, 183] and promotes progesterone synthesis [184]. In mouse models, IGF1 knock out leads to dwarfism and infertility with complete arrest in growing follicle pool and this phenomenon could not be rescued with gonadotropin stimulation [185]. Additionally, IGF1 has been identified in FF. In vitro cultured mature oocytes that successfully fertilized had more FF IGF1 expression compared to those that did not fertilize [186]. In human studies, FF IGF1 levels noted to correlate with follicle development, embryo quality and clinical pregnancy rates [187]. In the same study, estrogen levels noted to be higher in group with higher FF IGF1, supporting earlier finding of IGF1’s role in promoting aromatase activity [187].

Ovarian IGF production is affected by multiple factors. Similar to liver, GH promotes IGF1 production in GCs [188] and enhances FSH action with increasing FSH receptor expression [189]. Sequential treatment with GH and IGF1, promotes in vitro follicular growth with normal parameters of viability and ultrastructure [190]. DHEA supplementation in women undergoing IVF increases IGF1 expression especially in poor responders [191, 192].

Furthermore, IGF1 induces follicular angiogenesis by stimulating VEGF production within the follicle and promoting proliferation and differentiation of endothelial cells [107, 193].

After puberty, serum IGF1 expression was noted to decrease progressively [194, 195] (Fig. 2). To further explore IGF’s role in ovarian aging, Greenseid et al. examined IGF1 and IGF2 expression in GCs obtained from DOR patient and noted a significant decline in their expression compared to GCs obtained from control groups [181]. This was corroborated by Pashaiasl et al. who assessed key regulating genes in ovarian aging and noted downregulated expression of IGF2 in DOR patients [196]. Moreover, it has been observed that young women with DOR or older women compared to younger control, had significantly lower values of FF IGF1 [37, 197]. Interestingly, FF IGF1 levels were also used to assess clinical outcomes. For example, in young patients (24–35 years old) who obtained clinical pregnancy after single attempt IVF treatment compared to those who did not, FF IGF1 levels were shown to be higher [198]. Given IGF1’s role in aromatase activity and estrogen production, significantly downregulated IGF1 and IGF2 expression can explain suboptimal estrogen levels in the DOR population with gonadotropin stimulation during IVF treatment.

### Neurotropin family is a regulator of gonadotropin-independent and dependent stages of folliculogenesis and BDNF levels decrease within the aging follicles

Neurotropins are a family of growth factors, first described for their role in brain neurogenesis [199] and are identified as regulators of ovarian function in mammals, including humans [200–204]. The family is comprised of brain derived neurotropic factor (BDNF), nerve growth factor (NGF), neurotropin 3 (NT3), and neurotropin 4/5 (NT 4/5). They are known to have paracrine roles and support early germ cell survival, follicular development, steroidogenesis, oocyte maturation through polar body extrusion, and ovulation [204–211]. Among neurotropins, BDNF is the only one at present that has been demonstrated to be affected by ovarian aging [212].

BDNF has been identified in fetal and neonatal mammalian ovaries and plays role in various stages of folliculogenesis in adult ovaries [201, 203, 204, 207, 213–217]. Chow et al. demonstrated increasing BDNF levels with oogenesis in fetal growth [218]. BDNF expression also changes during the menstrual cycle and decreases with age and menopause [219]. It is important for oocyte maturation [214] and follicular maturation starting from the primordial stage. BDNF levels continuously increase while positively affecting *Gdf9* expression and GC proliferation rates [218]. In secondary follicles, BDNF upregulates *FSH-R* expression [218]. In human antral follicles, CC are responsible for BDNF secretion as evidenced by immunohistochemistry and in vitro studies [201, 203]. BDNF secreted by CC exerts its effect by binding to TrkB receptors located in the oocyte [201]. Its expression levels are upregulated following the LH surge [214]. In human CC treated with cAMP (whose expression also increases with exogenous gonadotropins), higher BDNF concentration was identified, though this wasn’t able to be replicated in GC or oocytes [201, 217]. In addition, BDNF was found in FF of antral follicles obtained from normally cycling women as well as in women after ovulation induction prior to IVF [201, 216] and the expression level was reflected by oocyte maturation rate [220]. Seifer et al. observed a higher level of BDNF expression in FF after ovulation induction compared to FF in natural cycles [216]. BDNF treatment of in vitro cultured mice oocytes had improved rate of maturation and first polar body extrusion [201, 214]. In mice, chronic stress was exhibited to reduce retrieved oocyte number, lower blastocyst formation rate and decrease BDNF expression in antral follicles; and treatment with BDNF was able to negate these effects [221]. In women with DOR and in endometriosis patients, FF BDNF levels obtained from antral follicles were observed to be lower [212]. With aging, especially after menopause, BDNF expression in follicles also noted to be downregulated significantly (Fig. 2). In a more recently published study, injection of agonistic TrkB inhibitor, Ab5B19, was able to attenuate the age related reduction in antral follicular count in vitro*,* indicating a possible role of BDNF in the follicular aging process [222].

## Potential modifiers of age-dependent changes in folliculogenesis

### CoQ10 levels decrease with age and CoQ10 supplementation may mitigate rate of decline of ovarian reserve with aging

ROS at physiological levels play an essential role in the female reproductive system [223]. When overabundant or imbalanced, ROS may lead to female infertility [224]. CoQ10 is a lipid soluble, natural antioxidant ubiquitously expressed in multiple organ systems including the cumulus oophorus complex and the FF [225]. It is an essential component of oxidative phosphorylation and ATP production in mitochondria and serves as a free radical scavenger with preventing lipid peroxidation and DNA oxidation functioning as an antioxidant [226]. In females, CoQ10 levels decrease with age [227] and levels in FF correlate with the quality of the oocyte contained within the follicle [225, 228]. In the aged oocyte, OXPHOS and ATP synthesis are noted to be reduced in human and mouse samples [229]. CoQ10 supplementation has been demonstrated to protect ovarian reserve from aging in rats and alleviate effects of aging in fertility outcomes [230]. In the aged mice model, CoQ10 treatment delayed ovarian reserve depletion and improved oocyte mitochondrial gene expression while improving mitochondrial activity [229]. This was correlated with an increase in ovulation and improvement in pregnancy rates [229]. One of the mechanisms by which CoQ10 ameliorates the effects of aging is by inhibiting DNA oxidation in mitochondria, thus inhibiting oocyte apoptosis [231]. As previously stated, post ovulatory oocytes were noted to have increased apoptosis and abnormal meiotic assembly [232]. Disrupted mitochondrial function and the increased DNA fragmentation rate in aged oocytes were alleviated with CoQ10 supplementation (Table 1). Negative impacts of mitochondrial dysfunction on meiotic spindle assembly and chromosomal misalignment were also improved with CoQ10 [229].

**Table 1:** List of ovarian follicular age-related modifiers. CoQ10, DHEA and Vitamin D can be used to mitigate age related changes in folliculogenesis and to improve fertility outcomes

*Supplemented Molecule*	*Effects*	*Reference*
CoQ10	alleviates aging related mitochondrial changes	[230]
	↑ mitochondrial gene expression and activity	[34, 229]
	↓ DNA oxidation	[231]
	delays ovarian primordial pool depletion	[229]
	↓ oocyte apoptosis	[231]
	↓ CC apoptosis	[34, 229]
	↑ oocyte quality	[34]
	↑ clinical pregnancy rates	[229]
DHEA	alleviates mitochondrial dysfunction	[133, 233–235]
	↑ energy production	[233]
	↑ response to ovarian stimulation	[192, 236–238]
	↑ peak estradiol level	[192, 236–238]
	↓ CC apoptosis	[192, 236–239]
	↑ oocyte quality	[233]
	↓ atretic follicle number	[192, 237, 238]
	↑ primordial recruitment	[240]
	↑ IGF1	[192, 241–244]
Vitamin D	↑ follicle growth and survival	[245, 246]
	↑ clinical pregnancy rates	[247]
	↑ oocyte maturation	[245, 246]
	↑ AMH production	[245, 246, 248, 249]

Furthermore, as reviewed earlier in this article, aging leads to a reduced number of cumulus cells surrounding the oocyte with increased apoptosis. This is accompanied by decreased expression of enzymes involved in CoQ10 synthesis in mice and human CCs [34]. Moreover, with aging, a notable reduction in the mitochondrial pool, glucose uptake [34], mitochondrial respiratory complex activity [250], and decreased expression of several OXPHOS genes are observed. Ben-Meir et al. was able to demonstrate reduced CC apoptosis and increased oocyte quality and quantity after oral CoQ10 supplementation in mice models [34, 229]. Administration of CoQ10 to the aged mice was able to improve mitochondrial metabolism, decrease apoptosis, restore CC number, stimulate glucose uptake, and increase progesterone synthesis [34]. Ben-Meir et al. was also able demonstrate improved mitochondrial OXPHOS gene expression after in vitro CoQ10 supplementation in aged human GCs [250]. In addition, in a systemic review and meta-analysis of women, five randomized control trials involving oral CoQ10 supplementation, it was concluded that oral supplementation of CoQ10 (in varying doses and durations: 600 mg daily for 8 weeks, 600 mg twice daily for 12 weeks or 200 mg three times daily for 8 weeks) increases clinical pregnancy rate with significant results in DOR patients (27.3% vs.17.5%; OR 1.83, 95% CI 1.04–3.24, *p* = 0.04; I2 0%) [251] (Table 1).

### Dehydroepiandrosterone supplementation may improve fertility in aged ovaries

DHEA is an essential prohormone, produced during synthesis of testosterone and estradiol from cholesterol by the zona reticularis layer of adrenal cortex and theca cells of ovary [252]. Its levels are observed to be high especially in early reproductive years and decline with age [253, 254]. Androgen receptors (AR) have been identified at several stages of folliculogenesis [255]. Androgens were shown to increase primary follicle number by activating primordial follicles in primate [256, 257] and mouse models [258]. They also have a role in follicle maturation and preovulatory follicle formation and by increasing *FSH-R* mRNA synthesis in mice and primate models [259, 260] and by enhancing GC proliferation demonstrated in in vitro [261] and GC specific AR knockout mouse models [262]. Global AR knockout mouse was shown to exhibit subfertility and defective folliculogenesis [263].

Similar to androgens, DHEA was demonstrated to be important for folliculogenesis, and administration of DHEA may improve IVF outcomes especially in populations with DOR or poor ovarian response. Casson et al. was among the first to describe beneficial effects of DHEA with DOR. They noted an increase in ovarian response to gonadotropin stimulation as well as increase in peak estradiol levels [192, 236]. Following Casson’s initial study, multiple mice and human studies with poor responders were conducted using various doses (10 mg – 80 mg per day) of DHEA administration for different durations of time (preIVF treatment or concurrently with ovarian stimulation) and noted improved ovarian function with increasing ovarian response while decreasing number of atretic follicles [192, 237, 238]. Li et al. exhibited increased oocyte quality and improved energy production in CCs of women older than 38 years who were pretreated with DHEA for at least 8 weeks prior to their IVF treatment [233]. In another study conducted by Sozen et al., a galactose induced premature ovarian insufficiency rat model with subfertility, decreased follicular number and increased atresia was used. DHEA was able to promote primordial follicular recruitment and stimulate follicular growth [240] (Table 1).

One of the mechanism that enables positive effects of DHEA was noted to be secondary to its role in increasing IGF1 which in turn may enhance response to gonadotropins and may have positive effects on oocyte quality, especially in poor responders [192, 241–244]. Furthermore, Zhang et al. corroborated improved BMP15 levels with 2-month of DHEA supplementation in poor ovarian response cases [264]. In addition, DHEA is noted to regulate AR expression and increase follicular recruitment and growth [262]. Another mechanism is attributed to DHEA’s effect in improving mitochondrial hemostasis and transport of oxidative phosphorylation and increasing cumulus cells mitochondrial oxygen consumption while shifting the energy production to aerobic metabolism from anaerobic metabolism which is commonly used in aging follicles [233, 234]. Its alleviating effect on preventing mitochondrial dysfunction with alternating expression of mitochondrial dynamics genes was also described by Wu [235] and Lin [133] (Table 1). PINK1 and PRKN, essential proteins for mitophagy, were noted to be downregulated and MFN1, a mitochondrial fusion gene expression was noted to be upregulated with DHEA treatment in human CCs [234]. DHEA also can slow down apoptosis of CCs of aging follicles [239]. The enhanced ovarian microenvironment with DHEA is hypothesized to decrease age related embryonic aneuploidy [265].

Testosterone is another androgen that can be used as a potential modifier of folliculogenesis. Within the ovary, it is produced theca layer of growing follicles [256]. It has been demonstrated promote FSH-R activity on GCs, leading to increase antral follicle response to gonadotropin stimulation [266]. In rhesus monkeys, testosterone administration was shown to promote primordial pool recruitment and increase primary follicular mass by inducing IGF1 production [257]. Same group also demonstrated increased preantral and small antral follicle numbers with testosterone supplementation in primates [256]. In human studies, baseline testosterone levels have been positively correlated with number of oocytes obtained after ovarian stimulation, although no direct correlation was noted with pregnancy outcome rates [267, 268]. Transdermal testosterone pretreatment on known poor responders was able to increase number of retrieved and fertilized oocytes, good quality embryos, and clinical pregnancy rates [269–271]. Reproductive aging has been associated with decreased serum testosterone levels [267, 272]. However, there is currently no available data assessing the impact of testosterone supplementation in ovarian aging mammalian models or among aged women undergoing IVF. Letrozole is an aromatase inhibitor which blocks conversion of testosterone to estrogen, thus leading to increase in testosterone levels. In poor responders, it has been demonstrated to increase number of retrieved oocytes and implantation rate [273] and decrease IVF costs through lowering gonadotropin dosage [274, 275]. However, there is insufficient data on its effect on clinical pregnancy or live birth rates [274, 276].

### Vitamin D level is positively correlated with reproductive potential and outcomes

Vitamin D (VD) is a fat-soluble secosteroid that is synthesized predominantly in the skin upon sunlight exposure but may also be obtained from dietary sources. It is metabolized in the liver to 25-hydroxyvitamin D, which is then metabolized in the kidneys by the enzyme 25-hydroxyvitamin D-1α-hydroxylase to its active form, 1,25-dihydroxyvitamin D [277]. This process is tightly regulated by plasma parathyroid hormone and serum calcium and phosphorus levels [277]. In target cells, VD binds to specific VD receptors (VDR) to regulate transcription of genes involved in diverse cellular processes, including pro-differentiation, anti-proliferation, pro-apoptosis, immunosuppression, and anti-inflammation roles [277]. Along with its essential role in bone physiology and health, there is an increasing recognition that VD is critical for normal folliculogenesis and optimizing reproductive potential in women [277, 278].

VD biosynthesis and signaling systems are expressed in ovarian follicles and detected in FF [245, 279–281]. In vitro studies on the effects of VD exposure on secondary preantral follicles isolated from rhesus monkeys revealed that VD supplementation improved follicle survival, growth, and function as well as oocyte maturation, and AMH production [245, 246]. They also confirmed that VD biosynthesis and signaling regulate follicular development in a stage-dependent manner, suggesting a potential endocrine and paracrine/autocrine actions of VD in the ovary and a stage-specific trophic effect on follicles [245, 246]. In goat and hen models, levels of VDR mRNA and protein expression correlate positively with antral follicle size [281, 282], suggesting that VD is involved in follicular maturation. VDR null mutant mice exhibit decreased aromatase activity in the ovary, resulting in impaired folliculogenesis [283]. Moreover, results of a study among Cyp27b1 (the rate-limiting enzyme in the synthesis of VD) null mice and wild-type mice randomized to VD-replete or -deficient diets supplemented with calcium, revealed that mice on VD-deficient diets had arrested follicular development and prolonged estrous cycles, with less oocytes retrieved from oviducts following gonadotropin stimulation [284].

VD levels have also been shown to be associated with ovarian reserve, as reflected in serum AMH levels. In premenopausal women with regular menstrual cycles, there was a correlation between circulating VD and AMH in women aged ≥40 years, suggesting that VD deficiency may be associated with lower ovarian reserve in late-reproductive-aged women [248]. In addition, VD acts as a regulator of *Amh* gene expression in vitro [249]. *AMH* gene was up-regulated by VD via functional VD response elements that bind the VDR in human prostate cancer cells [249]. Additionally, co-expression of steroidogenic factor 1, a key regulator of AMH, increased basal AMH promoter activity that was also found to be further stimulated by VD [249]. An inverse relationship between serum VD levels and ovarian reserve was also identified in patients with uterine fibroids [285]. Moreover, a *VDR* polymorphism among patients undergoing ovarian stimulation was correlated with decreased antral follicle counts [286] (Table 1).

VD levels also appear to affect reproductive potential and outcomes. In a study assessing infertile women undergoing IVF, higher FF levels of 25-hydroxyvitamin D were noted to be associated with significantly increased clinical pregnancy and implantation rates [287]. In several prospective studies, serum VD levels correlated positively with the number of mature oocytes retrieved and oocyte fertilization rates in patients undergoing the IVF [288–290]. In the same study, multivariable logistic regression analysis adjusting for age, BMI, ethnicity, and number of embryos transferred, identified FF levels of VD as an independent predictor of success of an IVF cycle [287]. In another study assessing a cohort of women undergoing IVF, the adjusted odds ratio for clinical pregnancy in women with vitamin D levels ≥20 ng/mL was significantly increased when compared to women with serum levels < 20 ng/mL [247]. In a subgroup analysis, it was concluded that women with the highest serum levels (> 30 ng/mL) had the highest chances of pregnancy [247]. However, studies assessing the prognostic value of FF VD on IVF outcomes have been inconsistent. Even though women with higher serum and FF vitamin D levels were found to be significantly more likely to achieve clinical pregnancy following IVF-embryo transfer in a prospective cohort study [283], another study noted lower quality embryos and significantly lower clinical pregnancy rates with higher levels of follicular VD levels [284].

## Summary

Forming an antral follicle that contains a healthy mature oocyte that is ready to be fertilized from a dormant primordial follicle is a well-orchestrated, complex process requiring exquisite synchronized timing of small intraovarian molecules and mitochondria. In this review, we summarized specific molecules and mechanisms that are crucial to the process of folliculogenesis and described how such molecules may be significantly impacted with aging. We then discuss well-studied modifiers, namely CoQ10, DHEA, and VD, that may be used to potentially improve fertility outcomes from aged follicles.

## Data Availability

Not applicable.

## Abbreviations

AFC: Antral follicle count
AMH: Antimullerian Hormone
AR: Androgen receptors
ART: Assisted reproductive technology
ATP: Adenosine triphosphate
BMP: Bone morphogenetic protein
BDNF: Brain derived neurotropic factor
CL: Corpus luteum
CC: Cumulus cells
CoQ10: Coenzyme Q10
Cyp19a1: Cytochrome P450 family 19 subfamily A member 1 (aromatase gene)
DHEA: Dehydroepiandrosterone
DOR: Diminished ovarian reserve
FF: Follicular fluid
FGF2: Fibroblast growth factor 2
FSH: Follicle-stimulating hormone
GC: Granulosa cells
GDF: Growth differentiating factor
GH: Growth hormone
IGF1: Insulin-like growth factor 1
IGFBP: Insulin-like growth factor binding protein
IL6: Interleukin 6
IUI: Intrauterine insemination
IVF: In vitro fertilization
LH: Luteinizing hormone
Lhcgr: Luteinizing hormone/choriogonadotropin receptor
mtDNA: Mitochondrial DNA
mtUPR: Mitochondrial unfolded protein response system
OXPHOS: Oxidative phosphorylation
NT: Neurotropin
ROS: Reactive oxygen species
Star: Steroidogenic acute regulatory protein
TNFa: Tumor necrosis factor alpha
VD: Vitamin D
VDR: Vitamin D receptor
VEGF: Vascular endothelial growth factor
